# Genetic Analysis of Sirtuin Deacetylases in Hyphal Growth of *Candida albicans*

**DOI:** 10.1128/mSphere.00053-21

**Published:** 2021-05-05

**Authors:** Guolei Zhao, Laura N. Rusche

**Affiliations:** a Department of Biological Sciences, State University of New York at Buffalo, Buffalo, New York, USA; University of Georgia

**Keywords:** *Candida albicans*, sirtuin, filamentous growth, nucleolar localization

## Abstract

Candida albicans is a major human fungal pathogen that encounters varied host environments during infection. In response to environmental cues, C. albicans switches between ovoid yeast and elongated hyphal growth forms, and this morphological plasticity contributes to virulence. Environmental changes that alter the cell’s metabolic state could be sensed by sirtuins, which are NAD^+^-dependent deacetylases. Here, we studied the roles of three sirtuin deacetylases—Sir2, Hst1, and Hst2—in the hyphal growth of C. albicans. We made single, double, and triple sirtuin knockout strains and tested their ability to switch from yeast to hyphae. We found that true hypha formation was significantly reduced by the deletion of *SIR2* but not *HST1* or *HST2*. Moreover, the expression of hypha-specific genes *HWP1*, *ALS3*, and *ECE1* decreased in the *sir2Δ/Δ* mutant compared to the wild type. This regulation of hypha formation was likely dependent on the deacetylase activity of Sir2, as a similar defect in hypha formation was observed when an asparagine known to be required for deacetylation was mutated. Finally, we found that Sir2 and Hst1 were localized to the nucleus, with Sir2 specifically focused in the nucleolus. This nuclear localization suggests a role for Sir2 and Hst1 in regulating gene expression. In contrast, Hst2 was localized to the cytoplasm. In conclusion, our results suggest that Sir2 plays a critical and nonredundant role in hyphal growth of C. albicans.

**IMPORTANCE** Candida albicans is one of the most common causes of hospital-acquired systemic fungal infections in the United States. It can switch between ovoid yeast and elongated hyphal growth forms in response to environmental cues. This morphological transition is essential for its survival in the host. Thus, identifying regulators involved in this process can lead to new therapies. In this study, we examined the contribution of three regulators called sirtuins (Sir2, Hst1, and Hst2) to the yeast-to-hypha transition of C. albicans. We found that loss of Sir2 but not Hst1 or Hst2 hampered hypha formation. Moreover, the defect was caused by the loss of the catalytic activity of Sir2. Our study may lay the groundwork for discovering novel targets for antifungal therapies.

## Abstract (precis)

Candida albicans is one of the most common causes of hospital-acquired systemic fungal infections in the United States. It can switch between ovoid yeast and elongated hyphal growth forms in response to environmental cues.

## INTRODUCTION

Candida albicans is a major opportunistic fungal pathogen of humans. In most healthy individuals, it is a harmless commensal living on mucosal surfaces and in the gastrointestinal tract. However, in immunocompromised people, it can cause severe superficial and life-threatening systemic infections. C. albicans accounts for up to 63% of all cases of candidemia, with a mortality rate of about 40% for systemic infections despite advanced antifungal treatment (1–5). Therefore, to develop new therapies, it is important to understand the mechanisms that contribute to the pathogenicity of C. albicans.

A defining virulence attribute of C. albicans is its ability to adopt several morphologies, including ovoid yeast cells, long chains of ellipsoid pseudohyphal cells, and parallel-sided true hyphal forms. The transition between yeast and hyphal forms is important for pathogenicity, since the hyphal form enables penetration into host organs, whereas the yeast form is adapted to dissemination through the bloodstream. Mutants that are defective in this morphological transition are attenuated in virulence in animal models of systemic infections (6–8). The morphological transition from yeast to hyphae is accompanied by the expression of many virulence factors. For example, adhesins, such as Als3 and Hwp1, mediate adherence of C. albicans to host cell surfaces (9, 10). In addition, secreted hydrolases, such as secreted aspartic proteases, facilitate active penetration into host cells (11). Moreover, the first identified cytolytic peptide toxin in C. albicans, Ece1, is highly expressed by hyphae and damages epithelial membranes (12, 13).

Other factors that assist in the transition from yeast to hyphae are transcription regulators, including deacetylases. For example, binding of the deacetylase Hda1 to the promoters of hypha-specific genes is required for hyphal maintenance by blocking the repressor, Nrg1 (14). In addition, the Set3/Hos2 deacetylase complex regulates morphogenesis of C. albicans by adjusting transcriptional kinetics of key morphogenic regulators (15). Finally, the deacetylase Rpd31 promotes hyphal extension, in part by activating transcription of the positive regulator *UME6* (16). These deacetylases are all classical deacetylases that deacetylate lysines using water to hydrolyze the acetyl group (17).

Sirtuins belong to a different family of deacetylases that couple lysine deacetylation to cleavage of NAD^+^, a coenzyme that is central to metabolism in all living cells. Sirtuins are found in organisms ranging from bacteria to humans, regulating numerous cellular processes, including transcription, metabolism, and cell aging (18–20). In pathogenic fungi, sirtuin deacetylases impact virulence. For example, in another human fungal pathogen Candida glabrata, the sirtuin Hst1 regulates genes required for antifungal resistance and oxidative stress response (21). In addition, the sirtuin Sir2 represses genes encoding adhesins, whose expression in low NAD^+^ promotes urinary tract infections (22). In C. albicans, there are five sirtuin-coding genes—*SIR2*, *HST1*, *HST2*, *HST3*, and *orf19*.*2963*—with similarity to Homo sapiens *SIRT5*. C. albicans Hst3, which deacetylates H3K56, is essential for normal hyphal growth, chromatin structure maintenance, and cell viability (23). Sir2 maintains the hypoacetylated state of the transcriptionally silent heterochromatin assembled at ribosomal DNA (rDNA) and telomeres (24–26). Sir2 may also affect phenotypic switching (27) and regulate the replicative life span of C. albicans in a dose-dependent manner, such that cells with more *SIR2* have extended lifespans (28). The role of C. albicans sirtuins has also been examined in the switch between two types of yeast cells called white and opaque. White cells are unable to mate, whereas opaque cells are mating competent (29). C. albicans lacking *HST1* had a reduced frequency of switching from the opaque to the white state, whereas cells lacking *HST2* had decreased switching from white to opaque (30). However, the functions of Sir2, Hst1, and Hst2 have not been well studied in the yeast-to-hypha morphological transition, an important virulence trait of C. albicans.

To study the roles of Sir2, Hst1, and Hst2 in the formation of hyphae, we made single-, double-, and triple-knockout strains using CRISPR (31) and compared the abilities of wild-type and knockout strains to form hyphae in two types of liquid media. In both yeast extract-peptone-dextrose (YPD) and spider media, hypha formation was significantly reduced in a *sir2* single mutant strain, but it was not affected by the deletion of *HST1*, *HST2*, or both. Double- and triple-deletion strains that lacked *SIR2* behaved similarly to the *sir2* single deletion, suggesting no redundant roles among the three sirtuins in hypha formation under the conditions tested. Moreover, the expression of the hypha-specific genes *HWP1*, *ALS3*, and *ECE1* decreased in the *sir2* mutant compared to the wild type. Furthermore, we found that the defect in hypha formation was likely due to the loss of Sir2 deacetylation activity, since it was disrupted by a point mutation of an amino acid required for catalysis. Finally, we found that Sir2 was localized to the nucleolus and Hst1 was localized to the whole nucleus, suggesting a role in regulating gene expression. In contrast, Hst2 was localized to the cytoplasm. In conclusion, our results suggest that Sir2 is important for hypha formation and thus for the virulence of C. albicans.

## RESULTS

### Sir2 is required for maximal true hypha formation.

**FIG 1:**
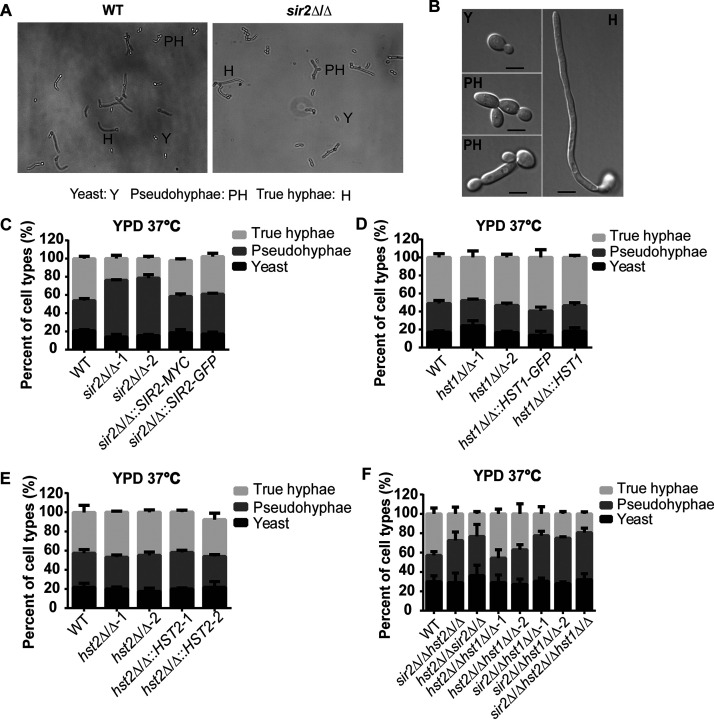
Sir2 deletion reduced true hypha formation in YPD at 37°C. (A) Three morphological forms of C. albicans were observed after growth at 37°C for 3 h. The ovoid budding yeast form (Y), the long chains of elongated ellipsoid pseudohyphae with constrictions at the septa (PH), and the parallel-sided true hyphae (H) are indicated. Images were taken at 20× magnification. (B) Higher-resolution images taken at 100× magnification showed the three distinct forms of C. albicans: yeast, pseudohyphae, and true hyphae. Scale bar, 5 μm. Homozygous *sir2*Δ/Δ (C), *hst1*Δ/Δ (D), or *hst2*Δ/Δ (E) single mutants, three double mutants, and the triple mutant (F) were grown at 30°C in YPD and then transferred to 37°C for 3 h. The percentages of each cell type were determined by counting at least 200 cells per strain. The mean percentages of cell types ± the standard errors (SE) of three independent experiments are shown.

To investigate the function of the three sirtuins, Sir2, Hst1, and Hst2, in the morphological transition of C. albicans, we used a recyclable CRISPR-Cas9 system (31) to construct mutant strains that included homozygous deletions of each sirtuin and all possible double and triple deletions. In addition, addback strains were made by incorporating the wild-type or epitope-tagged gene sequences into the genome of mutant strains at the endogenous loci. First, we tested whether the three sirtuins play roles in yeast-to-hypha transition triggered by elevating the temperature to 37°C (32). For this liquid filamentation assay, we inoculated C. albicans yeast-form cells grown overnight at 30°C into fresh YPD at 37°C and checked hypha formation microscopically after 3 h. The cell morphologies were divided into three categories as previously described (33): yeast, pseudohyphae, and true hyphae (Fig. 1A and B). We found that cells lacking Sir2 showed 1.5 to 2 times more pseudohyphae and half as many true hyphae compared to the wild type (Fig. 1C). This trend was also observed for the double and triple mutants in which *SIR2* was deleted (Fig. 1F). The percentages of yeast cells in all mutant strains were similar to that of the wild type, which is 20 to 30%. The percentages of true hyphae and pseudohyphae were restored to the wild-type levels by adding back *SIR2* at its original locus with either a Myc or green fluorescent protein (GFP) tag (Fig. 1C), indicating that the defect was due to loss of *SIR2*. In contrast to *SIR2*, deletion of *HST1* or *HST2* did not affect yeast-to-hypha transition under these conditions (Fig. 1D and E). Our results suggest that Sir2 is required for maximal true hypha formation when C. albicans cells are transferred from an overnight culture at 30°C into fresh YPD at 37°C.

**FIG 2:**
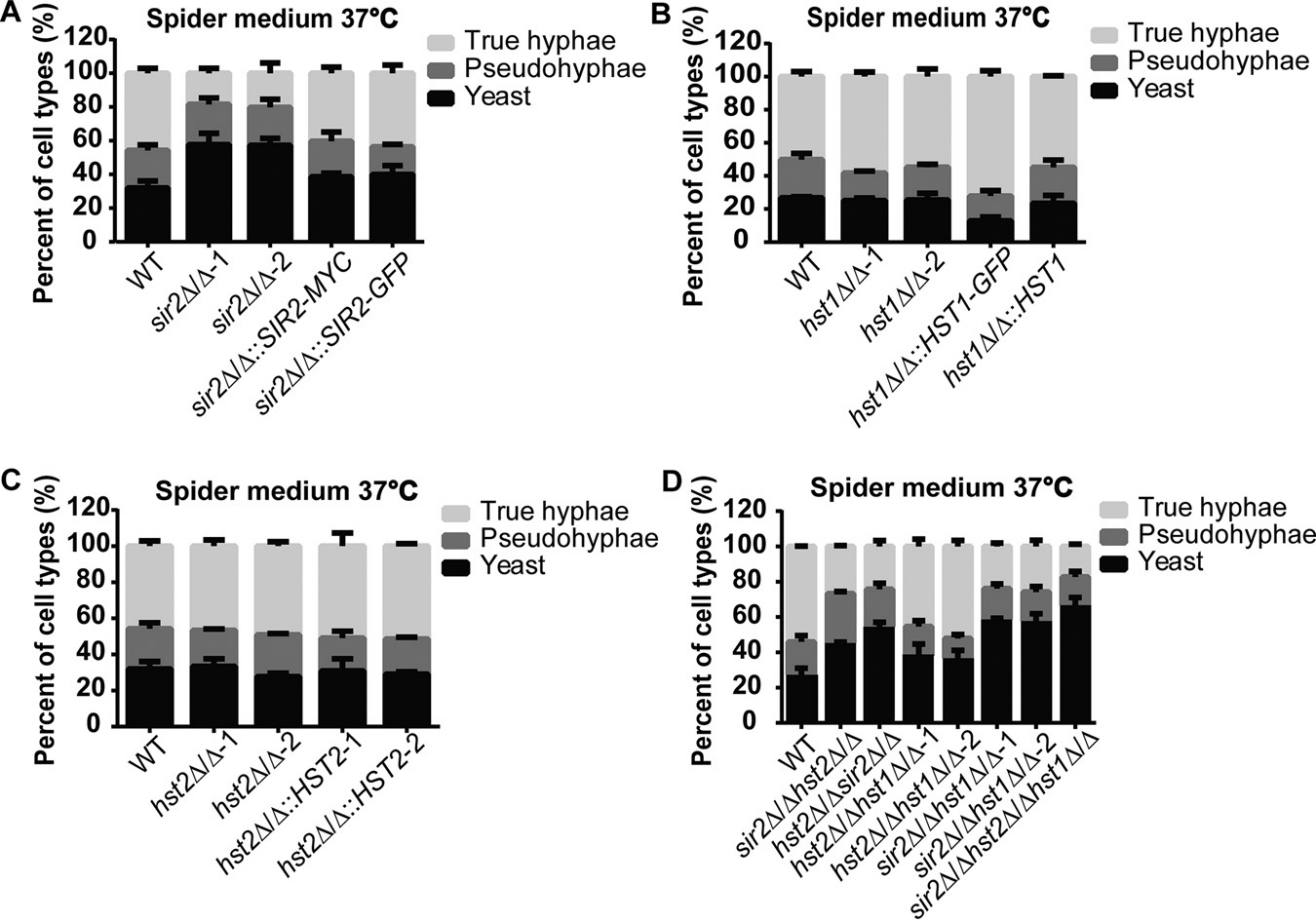
Sir2 deletion reduced true hypha formation in spider medium at 37°C. *sir2*Δ/Δ (A), *hst1*Δ/Δ (B), *hst2*Δ/Δ (C) single mutants, three double mutants, and the triple mutant (D) were grown at 30°C in YPD and then transferred to 37°C in spider medium for 3 h. The percentages of each cell type were determined by counting at least 200 cells per strain. The mean percentages of cell types ± the SE of three independent experiments are shown.

Although hyphae can be induced solely by elevating the temperature in media with the preferred carbon source, glucose, many widely used hypha-inducing media have poor carbon sources, such as mannitol in spider medium. To determine whether Sir2, Hst1, and Hst2 function in the morphological transition of C. albicans in media with a poor carbon source, we performed the liquid filamentation assay in spider medium at 37°C with the same mutant strains (Fig. 2). Similar to the results observed in YPD at 37°C, single, double, and triple mutants in which *SIR2* was deleted had half as many true hyphae compared to the wild type (Fig. 2A and D). In contrast to YPD, we found that in spider medium the strains lacking Sir2 had 1.5 to 2 times more yeast cells compared to the wild type (Fig. 2A and D), whereas the percentages of pseudohyphae in all strains were similar, which is around 20% (Fig. 2A and D). Adding back *SIR2* restored the percentages of true hyphae and yeast cells to the wild-type level (Fig. 2A). Comparable to the results observed in YPD at 37°C, the deletion of *HST1* and *HST2* did not affect yeast-to-hypha transition in spider medium at 37°C (Fig. 2B and C). Our results suggest that Sir2 is required for C. albicans yeast-to-hypha transition at 37°C in spider medium, which contains a nonfavorable carbon source.

### Expression of hypha-specific genes was affected by Sir2 deletion.

**FIG 3:**
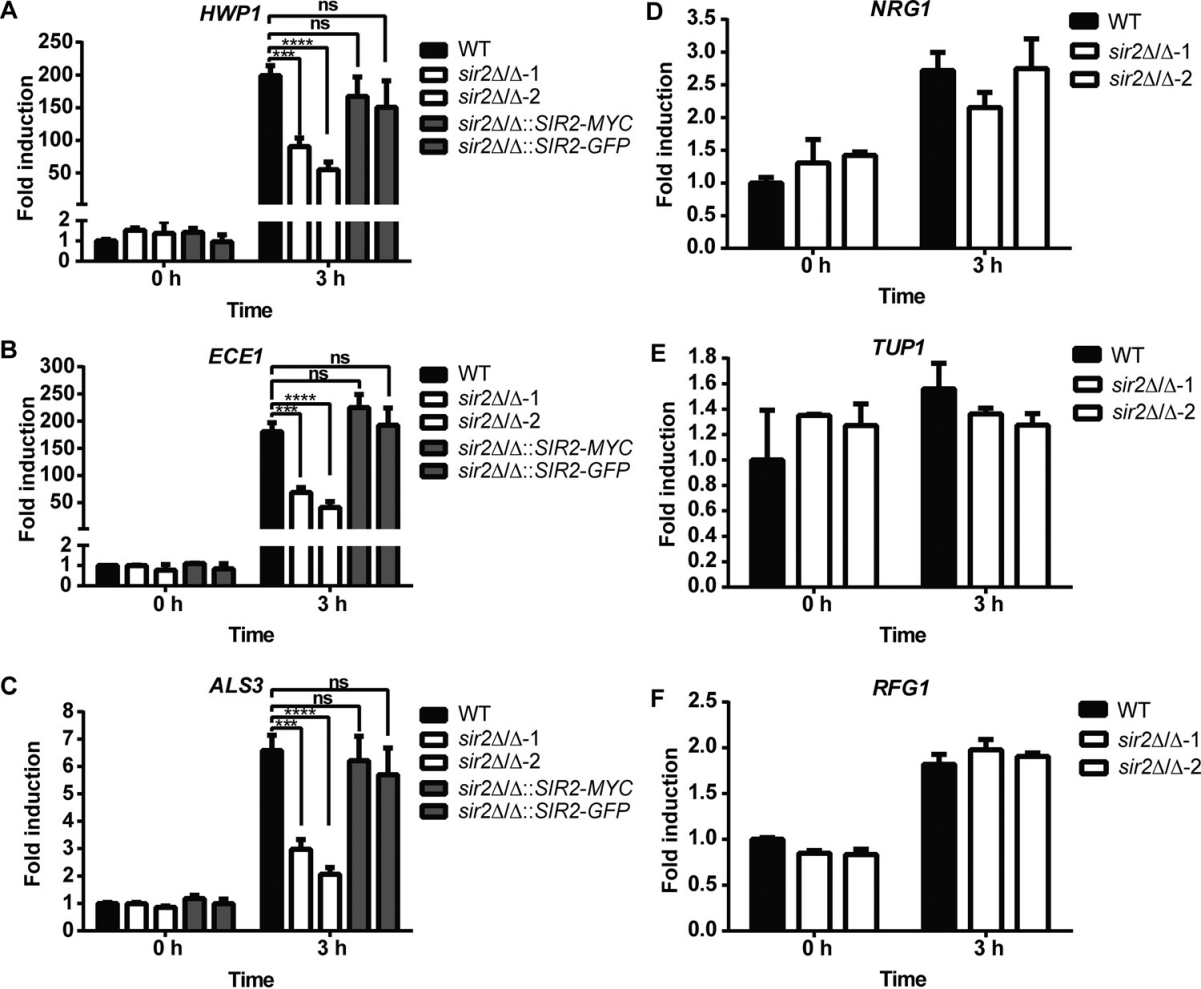
Expression of hypha-specific genes was reduced in Sir2 deletion strain. mRNA levels of the hypha-specific genes *HWP1* (A), *ECE1* (B), and *ALS3* (C) and the negative regulators of hyphal growth *NRG1* (D), *TUP1* (E), and *RFG1* (F) were measured by quantitative RT-PCR before (0 h-YPD, 30°C) and after (3 h-spider medium, 37°C) hypha induction. The fold induction was normalized to the wild type at time zero. The data represent the average of four independent replicates with error bars representing SEM. Asterisks show statistically significant differences (*, *P* < 0.05; **, *P* < 0.01; ***, *P* < 0.001; ****, *P* < 0.0001) based on two-way analysis of variance (ANOVA) with multiple comparisons.

C. albicans hypha formation is associated with the expression of hypha-specific genes (34, 35). *HWP1*, *ECE1*, and *ALS3* encode virulence factors and are highly expressed specifically in hyphal cells. As *sir2*Δ/Δ mutant had reduced true hypha formation, we tested whether the expression of hyphal-specific genes was affected. We analyzed the mRNA levels of *HWP1*, *ECE1*, and *ALS3* in the wild-type, *sir2*Δ/Δ, and the *SIR2* addback strains in spider medium at 37°C using reverse transcriptase followed by quantitative PCR (Fig. 3). In the absence of *SIR2*, cells expressed less *HWP1*, with a 2- to 3-fold difference between the wild-type and *sir2*Δ/Δ strains, consistent with a defective hyphal program (Fig. 3A). In addition, adding back *SIR2* with either a Myc or GFP tag restored *HWP1* expression to wild-type levels (Fig. 3A). Moreover, qualitatively identical results were obtained with *ECE1* (Fig. 3B) and *ALS3* (Fig. 3C). We performed a similar experiment on cells grown in YPD at 37°C, and we also detected decreased expression of *HWP1*, *ECE1*, and *ALS3* (see Fig. S1A to C in the supplemental material).

10.1128/mSphere.00053-21.1 FIG S1 The expression of hypha-specific genes was decreased, but the expression of negative regulators was not altered in YPD at 37°C. mRNA levels of hyphal-specific genes *HWP1* (A), *ECE1* (B), and *ALS3* (C) and negative regulators of hyphal growth *NRG1* (D), *TUP1* (E), and *RFG1* (F) were determined by quantitative RT-PCR before (0 h-YPD, 30°C) and after (3 h-YPD medium, 37°C) hypha induction. Fold induction was normalized to wild-type at time zero. The data represent the average of four independent replicates with error bars representing SEM. Asterisks show statistically significant differences (*, *P* < 0.05; **, *P* < 0.01; ***, *P* < 0.001; ****, *P* < 0.0001) based on two-way analysis of variance (ANOVA) with multiple comparisons. Download FIG S1, TIF file, 1.2 MB. Copyright © 2021 Zhao and Rusche. 2021 Zhao and Rusche. https://creativecommons.org/licenses/by/4.0/ This content is distributed under the terms of the Creative Commons Attribution 4.0 International license.

As expression of hypha-specific genes is repressed by Nrg1, Tup1, and Rfg1 (34, 36–39), it was possible that increased expression of these factors in *sir2*Δ/Δ strains inhibits filamentation. We therefore tested the expression of *NRG1*, *TUP1*, and *RFG1* in the same samples analyzed for hypha-specific gene expression. However, their expression did not change in the *sir2*Δ/Δ strain compared to the wild-type upon hyphal induction in spider medium (Fig. 3D to F) or YPD (see Fig. S1D to F). Taken together, our results suggest that Sir2 is required for yeast-to-hypha transition of C. albicans at both the cellular and the expression level.

### The defect in true hypha formation of *sir2*Δ/Δ cells is due to the loss of deacetylase activity.

**FIG 4:**
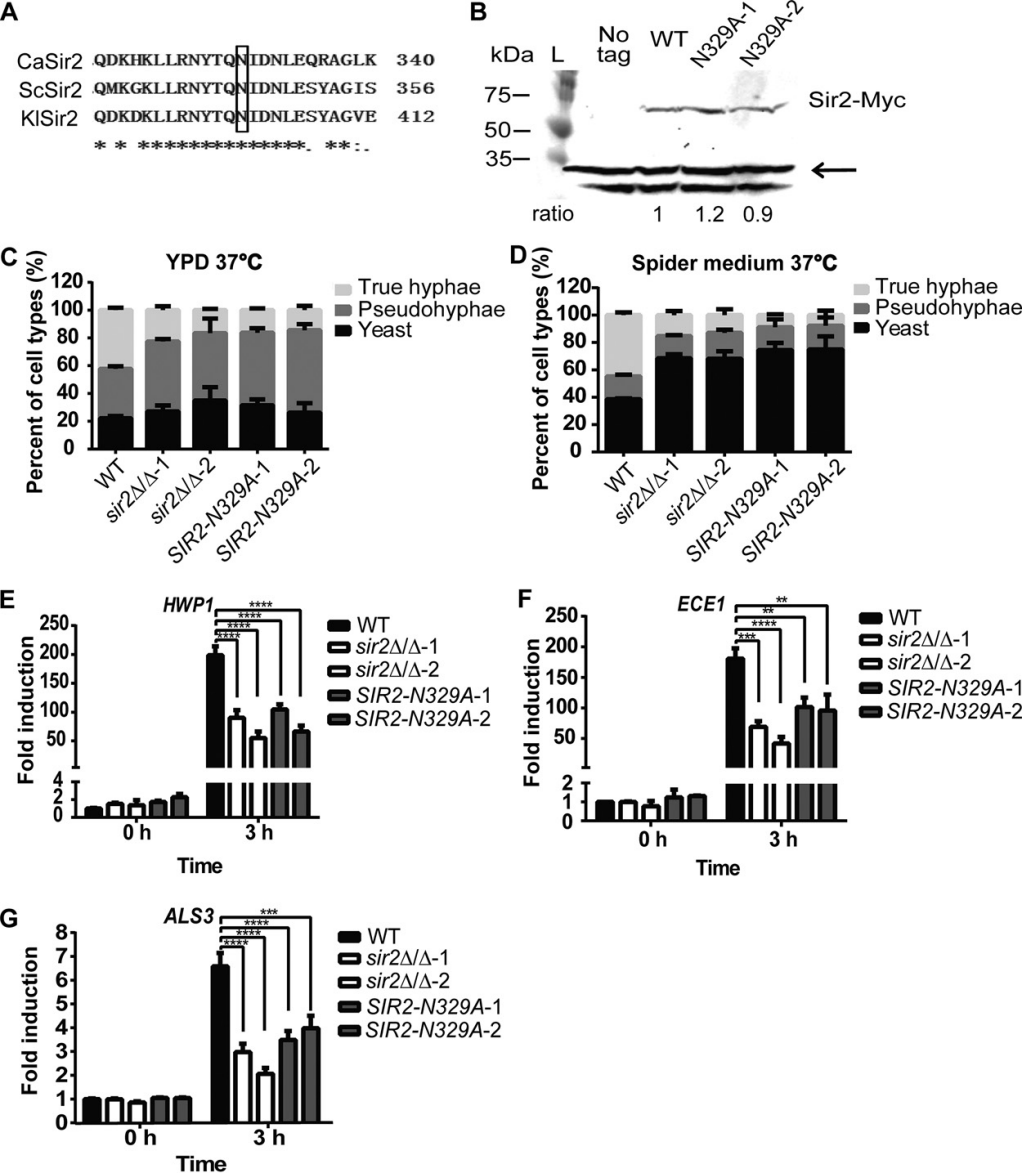
Mutation of a conserved asparagine in Sir2 required for deacetylase activity reduced true hypha formation. (A) Sequence alignment of C. albicans Sir2, S. cerevisiae Sir2, and K. lactis Sir2. The conserved asparagine (N) is boxed. (B) Myc-tagged wild-type Sir2 and Sir2-N329A were detected by immunoblotting using cell lysate from cells cultured in YPD at 30°C. A cell lysate prepared from an untagged wild-type strain was loaded as a control. Immunoblot results were quantified using ImageJ, and the expression levels of the Myc-tagged Sir2 were compared to the nonspecific bands indicated by the arrow. For each lane, the Sir2-Myc signal was divided by the Pgk1 signal. The ratios below the panels indicate the value for each lane compared to the value of the wild-type Sir2-Myc. (C and D) Abilities of *sir2*Δ/Δ and *sir2*-*N329A* strains to form hyphae were tested in YPD and spider medium at 37°C. The mean percentages of cell types ± the SE of three independent experiments are shown. (E, F, and G) Levels of *HWP1*, *ECE1*, and *ALS3* mRNA were measured by quantitative RT-PCR for the wild-type, *sir2*Δ/Δ, and the *sir2*-*N329A* strains before (0 h-YPD, 30°C) and after (3 h-spider medium, 37°C) hyphal induction. The data represent the average of four independent replicates, with error bars representing the SEM. Asterisks show statistically significant differences (*, *P* < 0.05; **, *P* < 0.01; ***, *P* < 0.001; ****, *P* < 0.0001) based on two-way ANOVA with multiple comparisons.

Sir2 and its homologous proteins are deacetylases that act on both histone and nonhistone substrates (40–43). To determine whether the deacetylase activity is required for hypha formation in C. albicans, we first identified a conserved asparagine residue that is required for deacetylase activity in Saccharomyces cerevisiae (44, 45). Based on an alignment of C. albicans Sir2 (CaSir2) with its homologs from S. cerevisiae (ScSir2) and Kluyveromyces lactis (KlSir2) (Fig. 4A) (46), we replaced the conserved asparagine residue (N329) in C. albicans Sir2 with alanine. Mutation of the equivalent asparagine in ScSir2 disrupts its deacetylase activity (44). To generate the catalytic mutant, the wild-type *SIR2* with a Myc tag was first reintroduced into the endogenous locus in the *sir2*Δ/Δ strain. Then, the N329A point mutation was introduced into the *SIR2*-*MYC* gene through CRISPR. The expression of Myc-tagged Sir2 and Sir2-N329A was similar as detected by immunoblotting (Fig. 4B). Compared to the reintroduced wild-type Sir2-Myc, which fully recapitulated the yeast-to-hypha transition in YPD and spider medium at 37°C (Fig. 1C and 2A), the *SIR2*-*N329A* point mutation resulted in fewer true hyphae in both media (Fig. 4C and D). The effect of this mutation is similar to that of the *sir2*Δ/Δ mutation. Further analysis of the expression of hypha-specific genes revealed that while the addback strain with wild-type Sir2 fully restored their expression (Fig. 3), strains expressing the Sir2-N329A point mutation displayed the same defective induction of hypha-specific genes as that in the *sir2*Δ/Δ strain (Fig. 4E, F, and G). Altogether, these data suggest that the defect in true hypha formation of *sir2*Δ/Δ is due to the loss of its deacetylase activity.

### Subcellular localization of Sir2, Hst1, and Hst2.

**FIG 5:**
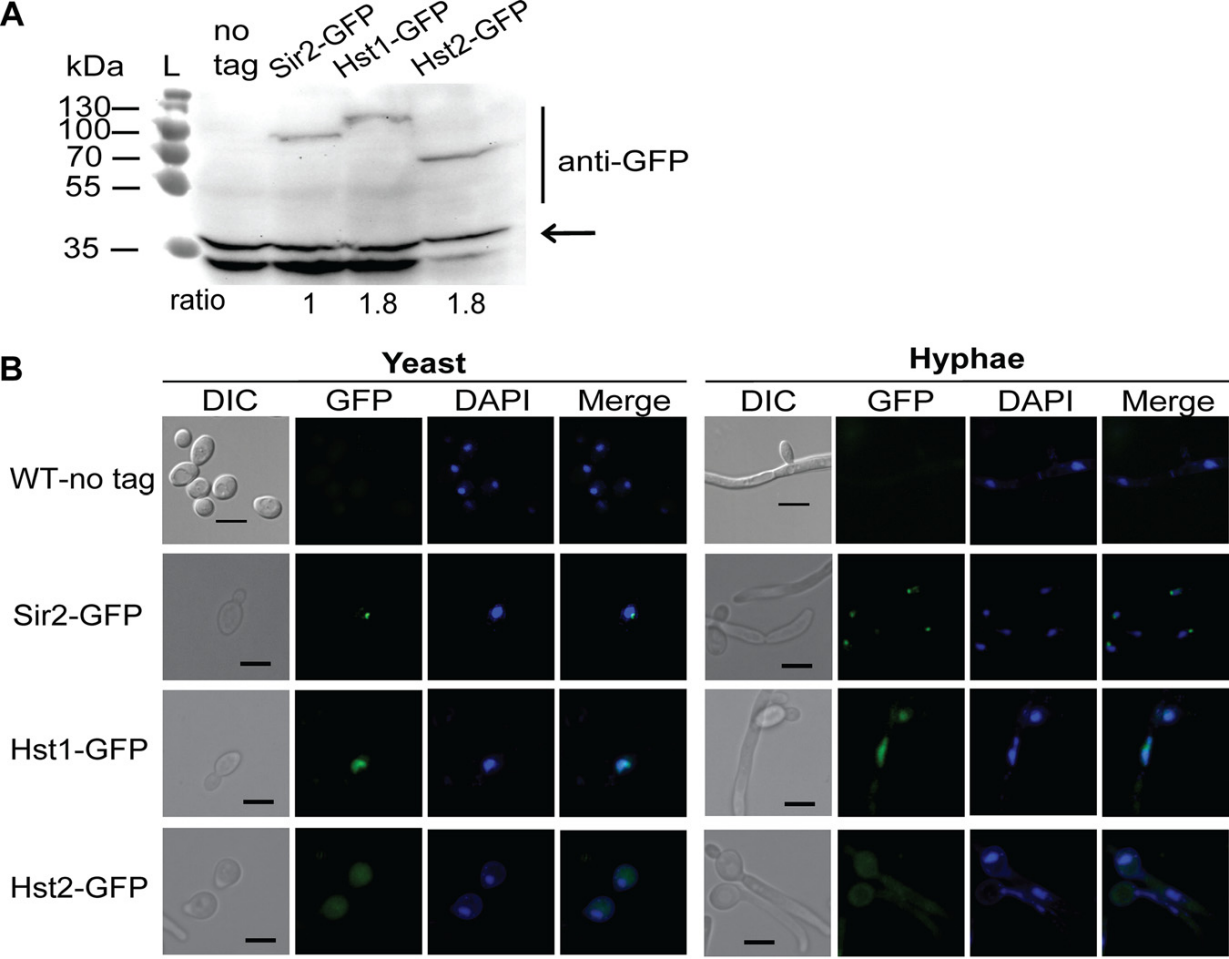
Subcellular localization of Sir2, Hst1, and Hst2. (A) Sir2-GFP, Hst1-GFP, and Hst2-GFP were detected by immunoblotting using cell lysate from cells cultured in YPD at 30°C. A cell lysate prepared from an untagged wild-type strain was loaded as a control. Immunoblot results were quantified using ImageJ, and the expression levels of the GFP-tagged sirtuins were compared to the nonspecific bands indicated by the arrow. (B) Cells were examined microscopically after growth in YPD at 37°C for 3 h. Subcellular localization of Sir2, Hst1, and Hst2 in both yeast cells and hyphae was indicated by the green fluorescence. DNA was stained with DAPI. Scale bar, 5 μm.

To narrow down the likely deacetylation targets of Sir2, Hst1, and Hst2, we investigated their subcellular distribution. To this end, we integrated *SIR2*-*GFP*, *HST1*-*GFP*, and *HST2*-*GFP* into the genomes of *sir2*Δ/Δ, *hst1*Δ/Δ, and *hst2*Δ/Δ strains at their endogenous loci. First, we confirmed the expression of the GFP-tagged Sir2, Hst1, and Hst2 by immunoblotting (Fig. 5A). All three sirtuins were successfully expressed under their native promoters. Furthermore, the GFP fusion did not interfere with the function of Sir2, since the *SIR2-GFP* addback strain restored true hypha formation and hyphal gene expression to the wild-type level (Fig. 1C, 2A, and 3). Next, we examined the subcellular localization of the three sirtuins in both yeast cells and hyphae using fluorescence microscopy (Fig. 5B). The wild-type strain with no *GFP* integration was used as a negative control for background fluorescence. For Sir2-GFP, the fluorescence was concentrated in a discrete part of the nucleus both in yeast cells and hyphae. In contrast to Sir2-GFP, Hst1-GFP was distributed throughout the nucleus in both yeast and hyphal cells. Finally, Hst2-GFP was found throughout the cell, in some cases appearing to be excluded from the nucleus. Thus, the three sirtuins have distinct subcellular localizations.

**FIG 6:**
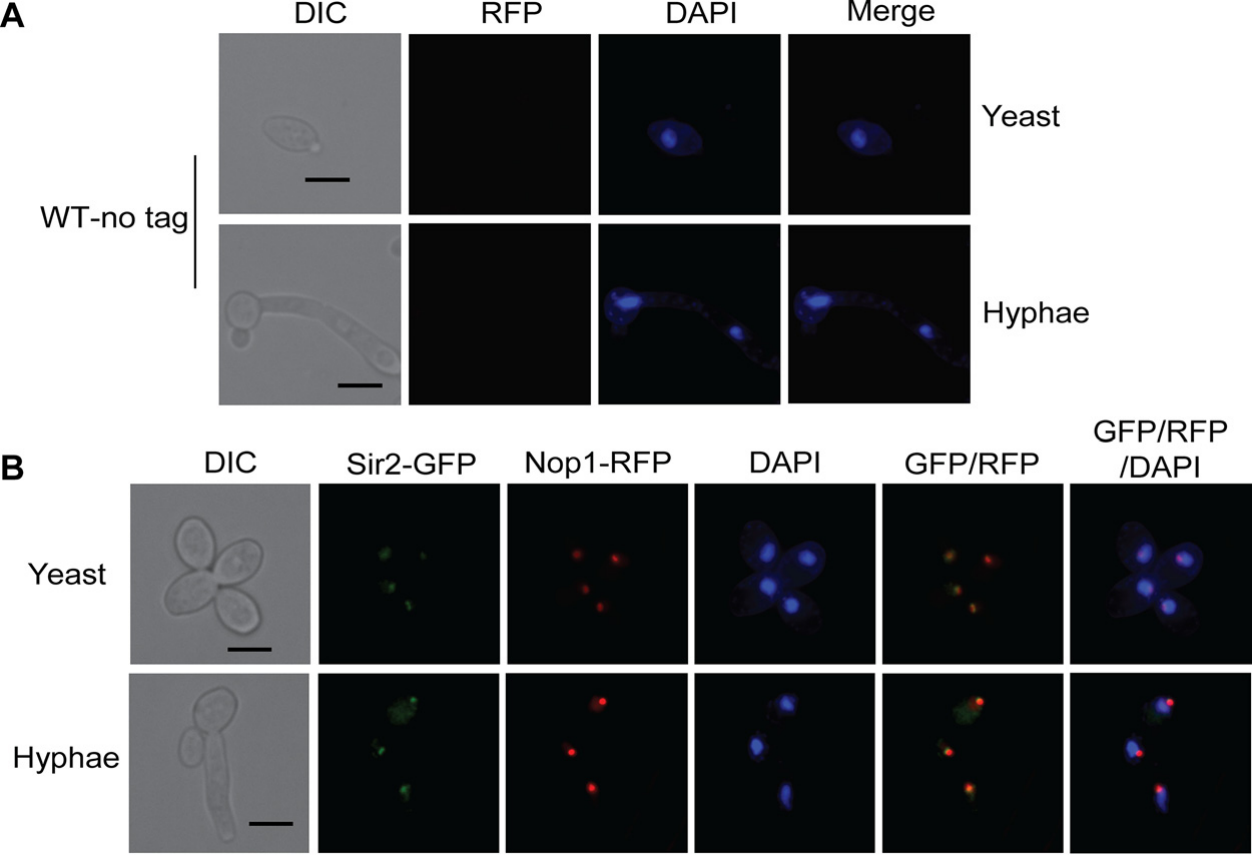
Sir2 colocalized with nucleolar protein Nop1. Cells were examined microscopically after growing in YPD at 37°C for 3 h. Localization of Sir2 and Nop1 were indicated by the green and red fluorescence, respectively. DNA was stained with DAPI. Scale bar, 5 μm.

In S. cerevisiae, Sir2 is concentrated in the nucleolus, where it regulates recombination between the rDNA repeats (47). A similar role is expected for CaSir2, which deacetylases histones within the rDNA array (25, 26). To determine whether the subnuclear focus we observed for Sir2-GFP coincides with the nucleolus, we tagged the nucleolar protein Nop1 with red fluorescent protein (RFP) (Fig. 6). The wild-type strain with no *RFP* integration was used as negative control and did not show any red fluorescence (Fig. 6A). We found that Sir2-GFP colocalized with Nop1-RFP, suggesting that Sir2 is localized to the nucleolus (Fig. 6B). Together, our results suggest that both Sir2 and Hst1 are nuclear proteins, whereas Hst2 is a cytoplasmic protein. These localization patterns are consistent in yeast and hyphal cells. These different localization patterns indicate distinct functions for Sir2, Hst1, and Hst2 in C. albicans, which may explain their different roles in filamentation.

## DISCUSSION

In this study, we explored the roles of three sirtuins, Sir2, Hst1, and Hst2, in the hyphal induction of C. albicans. We made single, double, and triple mutants of the sirtuins and tested their ability to form hyphae. Our liquid filamentation assay indicated the importance of Sir2, but not Hst1 or Hst2, in hypha formation at 37°C. Consistent with the defect in hyphal formation in the *sir2* mutant, the expression of hypha-specific genes decreased in the absence of *SIR2*. Moreover, the defect in hypha formation in the *sir2* mutant resulted from the loss of the deacetylase activity of Sir2. We found that the contribution of Sir2 to hyphal growth was not achieved through regulating expression of the negative regulators of hyphal growth. Our study of morphological transitions is relevant to the virulence of C. albicans, since the yeast-to-hypha transition in liquid media would mimic filamentation of C. albicans in bodily fluids or within phagocytic cells. Finally, we recorded the subcellular localization of the three sirtuins. Sir2 is mainly localized to the nucleolus, as was previously observed in S. cerevisiae (47, 48). This finding is consistent with its role in regulating the rDNA tandem repeats (25, 26). However, given the silencing function of C. albicans Sir2 at subtelomeres (24–26), Sir2 may also be present at lower, less detectable levels in the rest of the nucleus and regulate factors involved in filamentation. We also observed the nuclear localization of Hst1 and the cytoplasmic localization of Hst2. The presence of Sir2 and Hst1 in the nucleus suggests a role in regulating gene expression.

Our results differ from those presented previously (27), which showed that the deletion of *SIR2* resulted in spontaneous filamentation and wrinkled colony morphologies, with a high frequency of switching among colony morphologies. In contrast, we report reduced filamentation and did not observe wrinkled colonies when we streaked our *sir2*Δ/Δ strains. These differences might arise from different growth conditions or strains. We note that we observed the same phenotype for two independent deletions, and the phenotype reverted to wild-type when we added back the *SIR2* gene.

Our study revealed that expression of hypha-specific genes decreased in the absence of Sir2. This finding likely reflects the reduced number of hyphae because these genes are only expressed in hyphal cells. Moreover, these genes are unlikely to be the direct targets of Sir2 because direct targets are expected to increase in expression in the absence of histone deacetylation by Sir2. It was theoretically possible that Sir2 directly regulates genes that repress filamentation and that increased expression of these genes causes reduced filamentation. However, the expression of three negative regulators, *NRG1*, *TUP1*, and *RFG1*, did not change in the absence of Sir2. Nevertheless, our results indicated that the contribution of Sir2 to hyphal induction depends on its deacetylase activity. Thus, the hypha-specific genes are likely indirectly influenced by Sir2 through its regulation of other genes or proteins, and it will be necessary to identify these direct genomic and protein targets of Sir2 to understand the mechanism by which Sir2 promotes hyphal induction. Given that the rDNA locus is deacetylated by Sir2 in C. albicans (25, 26), presumably to suppress unbalanced recombination as in S. cerevisiae (49, 50), one possibility is that the defect in hypha formation in the *sir2* mutant is a result of unbalanced recombination within the tandem rDNA array. Consistent with this idea, in Candida parapsilosis changes in colony morphology are accompanied by size alterations of the chromosome containing the rDNA gene cluster (51). Another possibility is that Sir2 promotes hyphal growth through regulating the expression of telomere-associated (*TLO*) genes, which can enhance filamentation and resistance to oxidative stress (24, 52, 53). It is also possible that Sir2 regulates expression of hypha-specific genes by deacetylating nonhistone proteins. A well-studied example of such regulation is the deacetylase Hda1, which helps maintain hyphal development in C. albicans (14). In the initiation phase of hyphal development, the NuA4 histone acetyltransferase complex binds to the promoters of hypha-specific genes to activate their transcription. However, in the maintenance phase, the deacetylase Hda1 removes the NuA4 complex from these promoters by deacetylating the subunit Yng2, thereby allowing reassembly of nucleosomes that block the repressor Nrg1. Therefore, to understand the role of Sir2 in regulating hyphal growth, it will be necessary to identify the protein targets of Sir2.

Hyphal growth can be triggered by a variety of host environmental conditions that C. albicans encounters during infection. These include elevated temperature, nitrogen starvation, serum, CO_2_, and alkaline pH (32). However, factors involved in sensing these environmental cues still need to be uncovered. Since the deacetylation activity of Sir2 requires NAD^+^, which is a key redox carrier in central metabolism, environmental conditions that impact intracellular [NAD^+^] may be interpreted by Sir2 and passed on to its downstream targets that influence filamentation. For example, changes in metabolic flux due to nutrient perturbations could impact NAD^+^ availability. In disseminated candidiasis, C. albicans gains access to the bloodstream, where glucose is plentiful (47). When glucose is oxidized for energy through glycolysis, NAD^+^ is reduced to NADH. However, if C. albicans is engulfed by macrophages or neutrophils, the environment has less optimal nutrient sources, such as lipids and amino acids (33). These nonsugar compounds enter metabolic pathways that require glycolysis to run in reverse (gluconeogenesis). Thus, yeast may reach different steady-state [NAD^+^]/[NADH] ratios depending on the available carbon sources, which could in turn impact sirtuin activity. Indeed, in another pathogenic yeast, C. glabrata, NAD^+^ levels regulate expression of virulence factors through Sir2 and Hst1 (21, 22). In addition, in the yeast Kluyveromyces lactis, NAD*^+^* and Sir2 regulate stress response genes that mitigate the effects of oxidative stress and DNA damage (27). Therefore, sirtuins help yeast species sense stresses and activate appropriate responses. Thus, our study is consistent with C. albicans using Sir2 to modulate filamentation in response to environmental cues during infection.

Our study also indicates that Sir2, Hst1, and Hst2 have distinct roles in C. albicans, even though Sir2 and Hst1 are paralogs resulting from an ancient gene duplication. This result is in agreement with previous studies that supported the distinct roles of Sir2 and Hst1 in C. albicans (26). For example, silencing at the rDNA locus and subtelomeres is performed by Sir2 rather than Hst1 (26). Hst2 is evolutionarily more divergent from Sir2 and Hst1 (54), and thus its function is expected to be different. For instance, in S. cerevisiae, Hst2 cannot complement the silencing deficiency caused by Sir2 deletion (47). In addition, the different subcellular localizations of Sir2, Hst1, and Hst2 observed in this study also suggested their distinct functions. Our study did not provide evidence that Hst1 and Hst2 function in filamentation of C. albicans, so their functions remain to be uncovered.

In this study, we explored the roles of three sirtuins in hyphal growth of C. albicans. We found that Sir2 is critical for hypha formation and moreover, its function in hypha formation depends on its deacetylase activity. Since morphological plasticity is an important virulence trait of C. albicans, our study lays the groundwork for discovering novel targets for antifungal therapies.

## MATERIALS AND METHODS

### Media and growth conditions.

C. albicans strains were stored in 25% glycerol stocks at −80°C. For routine manipulation during strain construction, strains were grown in YPD (1% yeast extract, 2% peptone, 2% glucose) at 30°C. Transformants were selected on YPD with 0.2 mg/ml nourseothricin (ClonNAT). Cells in which the CRISPR cassette had been excised were selected on CSM-leucine (0.67% yeast nitrogen base without amino acids, 2% glucose, 0.69 g/liter CSM-Leu powder [Sunrise Biosciences, catalog no. 1005-100]). For filamentation assays, cells were grown in YPD or spider medium (1% mannitol, 1% nutrient broth, 0.2% K_2_HPO_4_ [pH 7.2]) at 37°C.

### Plasmid construction.

To make plasmids (Table 1) that contain repair templates for tagging genes, we first amplified *SIR2* (C2_01330C), *HST1* (C1_09050W), *HST2* (CR_01800C), and *NOP1* (C4_06720W) with ∼500 bp upstream and downstream of the reading frame using primers with restriction sites (Table 2). The PCR products were then digested and ligated into plasmid pRS414 (55). We next amplified *GFP* (pLR1221), *RFP* (yEpGAP-Cherry), or *MYC* (pWZV87) tags from plasmids using primers whose 5′ sequences matched the intended insertion sites in the genes to be tagged (Table 2). Finally, these tags were incorporated at the 3′ ends of the target genes through PCR stitching (17 cycles of 40 s at 95°C, 50 s at annealing temperature specified by the New England Biolabs *T_m_* calculator, and 20 min at 68°C). Correct stitching of the tag was confirmed by Sanger sequencing.

**TABLE 1:** Plasmids used in this study

Plasmid	Description	Source or reference
pADH110	Universal template for fragment A for cloning-free stitching of gRNA expression cassette	31
pADH119	C. albicans LEUpOUT template for fragment B for cloning-free stitching of gRNA expression cassette	31
pADH137	C. albicans LEUpOUT CAS9 expression plasmid	31
pRS414	Empty vector used to make repair template for GFP-, Myc-, or RFP-tagged genes	55
pWZV87	*MYC*	58
pAE28	pRS316 with centromere 3 of Torulaspora delbrueckii	Jasper Rine
pLR1221	*GFP* in pAE28	This study
yEpGAP-Cherry	*RFP*	59
pLR1257	*HST1* with flanking sequence	This study
pLR1260	*SIR2* with flanking sequence	This study
pLR1268	*SIR2*-*MYC*	This study
pLR1269	*SIR2*-*GFP*	This study
pLR1271	*HST1*-*GFP*	This study
pLR1309	*HST2* with flanking sequence	This study
pLR1310	*HST2*-*GFP*	This study
pLR1311	*NOP1* with flanking sequence	This study
pLR1312	*NOP1*-*RFP*	This study

**TABLE 2:** Oligonucleotides for amplifying genes and tags for cloning

Description	Sequences^*a*^
*GFP* with flanking SpeI/SacI sites	F: ACTAGTTCTAAAGGTGAAGAATTATTC R: GAGCTCTTTGTACAATTCATCCATACCATGGG
*HST1* with flanking SacII/SacI sites	F: GCGCGCCGCGGGAAAAGTTCACCCCCCTCAC R: GCGCGGAGCTCGGTGGTAGTAGCAACATAGC
*SIR2* with flanking SpeI/SacII sites	F: GCGCGACTAGTGGTAGTGGGTCACAAACAATAAG R: GCGCGCCGCGGGGTGGTATCTTGGATTCAACC
*HST2* with flanking SalI/SacII sites	F: GCGCGGTCGACCCTCTGTTGAGATTGTCACG R: GCGCGGAGCTCGCTTTAGGCTATTCATCCCATC
*MYC* to be integrated at the 3′ end of *SIR2*	F: GGGAAATTGTCAAGAAATCAACTTCGACAAAAAAAGCTGCTAGTGGTGAACAAAAG R: CACAAAGATACCCAAACTCCTATCTCAGGATCCGTTCAAGTCTTCTTC
*GFP* to be integrated at the 3′ end of *SIR2*	F: GGGAAATTGTCAAGAAATCAACTTCGACAAAAAAATCTAAAGGTGAAGAATTATTC R: CACAAAGATACCCAAACTCCTATCTCATTTGTACAATTCATCCATACCATGGG
*GFP* to be integrated at the 3′ end of *HST1*	F: CGAAAGTGGAGGTAAAAGTGGATCTAAAGGTGAAGAATTATTC R: CTATCGGGGCTTTCTCTTCCTCATTTGTACAATTCATCCATACCATGGG
*GFP* to be integrated at the 3′ end of *HST2*	F: CAAAGAATTAGAACAATTGATAGATAAATTAAAAATTTCTAAAGGTGAAGAATTATTC R: CTTGAAAATGTATATTATATTTTTGTTGAGACTCATTTGTACAATTCATCCATACCATGGG
*NOP1* with flanking SpeI/SacII sites	F: GCGCGACTAGTCTAACATCTTGGTCATGGCTC R: GCGCGCCGCGGCACATCCAATGAAACTTCTCGTG
*RFP* to be integrated at the 3′ end of *NOP1*	F: GAGAAGCGGAATAAAGAAAATGGTTTCAAAAGGTGAAGAAGATAATATGG R: GAGTATCCCAAAATAACCTCATTTATATAATTCATCCATACCACCAGTTG

a *SIR2*, *HST1*, *HST2*, and *NOP1* were amplified with primers containing restriction sites (underlined). *MYC*, *GFP*, and *RFP* were amplified with chimeric primers that annealed to the tag (underlined) and the gene into which it was inserted (not underlined).

To create plasmid pLR1221 bearing the *GFP* gene, we started with a C. albicans Sir2-GFP strain (YJB13051, Judith Berman). *GFP* was amplified from YJB13051 and ligated into the SpeI and SacI sites of pAE28 (Aisha Ellahi and Jasper Rine), yielding pLR1209. Finally, the sequence encoding three amino acids in GFP (L64 [TTC>TTA], T65 [GGT>ACT], and S72 [GCG>TCT]) was corrected by site-directed mutagenesis to generate pLR1221.

### Strain construction.

C. albicans strains (Table 3) were constructed using a *LEU2*/*leu2* derivative of SC5314 to facilitate CRISPR-mediated genome editing using described procedures (31). Briefly, a CRISPR “LEUpOut” cassette expressing Cas9, a specific guide RNA, and a nourseothricin (ClonNAT) resistance gene was assembled by PCR stitching and homologous recombination. This cassette, which was flanked by fragments of *LEU2*, was integrated at the *LEU2* locus, generating a strain that was auxotrophic for leucine and resistant to nourseothricin. Cells were simultaneously transformed with two halves of the CRISPR LEUpOut cassette and a repair template directed to the same locus as the guide RNA. For each transformation, a minimum of 1 μg of repair template and 2 μg of CRISPR cassette was used. After confirming by PCR that the desired genome editing had occurred, cells were plated on medium lacking leucine to select for the excision of the CRISPR cassette from the *LEU2* locus.

**TABLE 3:** Yeast strains used in this study

Strain	Genotype	Source or reference
AHY940	SC5314 *LEU2*/*leu2*Δ (used as wild-type in this study)	31
YJB13051	*SIR2*-*GFP*::*URA3*/*SIR2*-*GFP*::*HIS1 URA3*/*URA3 his1*::*HISG*/*his1*::*HISG*	Judith Berman
LRY3225	AHY940 *sir2Δ*/*sir2Δ*-1	This study
LRY3226	AHY940 *sir2Δ*/*sir2Δ*-2	This study
LRY3235	AHY940 *sir2Δ*::*SIR2-GFP*/*sir2Δ*::*SIR2-GFP*	This study
LRY3236	AHY940 *sir2Δ*::*SIR2-MYC*/*sir2Δ*::*SIR2-MYC*	This study
LRY3366	AHY940 *sir2Δ*::*SIR2*(*N329A*)*-MYC*/*sir2Δ*::*SIR2*(*N329A*)*-MYC*-1	This study
LRY3367	AHY940 *sir2Δ*::*SIR2*(*N329A*)*-MYC*/*sir2Δ*::*SIR2*(*N329A*)*-MYC*-2	This study
LRY3237	AHY940 *hst1Δ*/*hst1Δ*-1	This study
LRY3238	AHY940 *hst1Δ*/*hst1Δ*-2	This study
LRY3239	AHY940 *hst1Δ*::*HST1*-*GFP*/*hst1Δ*::*HST1*-*GFP*	This study
LRY3368	AHY940 *hst1Δ*::*HST1*/*hst1Δ*::*HST1*	This study
LRY3231	AHY940 *hst2Δ*/*hst2Δ*-1	This study
LRY3232	AHY940 *hst2Δ*/*hst2Δ*-2	This study
LRY3233	AHY940 *hst2Δ*::*HST2*/*hst2Δ*::*HST2*-1	This study
LRY3234	AHY940 *hst2Δ*::*HST2*/*hst2Δ*::*HST2*-2	This study
LRY3357	AHY940 *hst2Δ*::*HST2-GFP*/*hst2Δ*::*HST2-GFP*	This study
LRY3241	AHY940 *sir2Δ*/*sir2Δ hst2Δ*/*hst2Δ*	This study
LRY3242	AHY940 *hst2Δ*/*hst2Δ sir2Δ*/*sir2Δ*	This study
LRY3243	AHY940 *sir2Δ*/*sir2Δ hst1Δ*/*hst1Δ*-1	This study
LRY3244	AHY940 *sir2Δ*/*sir2Δ hst1Δ*/*hst1Δ*-2	This study
LRY3245	AHY940 *hst2Δ*/*hst2Δ hst1Δ*/*hst1Δ*-1	This study
LRY3246	AHY940 *hst2Δ*/*hst2Δ hst1Δ*/*hst1Δ*-2	This study
LRY3247	AHY940 *sir2Δ*/*sir2Δ hst2Δ*/*hst2Δ hst1Δ*/*hst1Δ*	This study
LRY3451	AHY940 *NOP1*-*RFP*/*NOP1*	This study
LRY3453	AHY940 *sir2Δ*::*SIR2-GFP*/*sir2Δ*::*SIR2-GFP NOP1*-*RFP*/*NOP1*	This study

To delete *SIR2*, *HST1*, and *HST2*, either singly or in combination, we used 20-bp guide RNAs (Table 4) targeting the open reading frame of each gene. The repair templates (Table 5) were generated using oligonucleotides with 49 bases of homology both upstream and downstream of the target gene. A GG dinucleotide was introduced at the junction between the upstream and downstream sequences to function as PAM for a guide RNA in a subsequent gene addback. To create a double-stranded repair template, two complementary oligonucleotides were heated to 99°C for 30 s and then ramped down to 65°C at a rate of 0.1°C/s.

**TABLE 4:** Oligonucleotides used to create gRNAs^*a*^

Mutation	Oligonucleotide used to create guide RNA
*hst1*Δ	CGTAAACTATTTTTAATTTGGGACAACGACGAAGAGGAAGGTTTTAGAGCTAGAAATAGC
*sir2*Δ	CGTAAACTATTTTTAATTTGGCTACCACCACGCCTACTGCGTTTTAGAGCTAGAAATAGC
*hst2*Δ	CGTAAACTATTTTTAATTTGATACGGGTCTTTATGCAAACGTTTTAGAGCTAGAAATAGC
*HST1* addback	CGTAAACTATTTTTAATTTGAATAACAAATAACAATCAATGTTTTAGAGCTAGAAATAGC
*SIR2* addback	CGTAAACTATTTTTAATTTGTTTGAGAGAAATCCTCTAGTGTTTTAGAGCTAGAAATAGC
*HST2* addback	CGTAAACTATTTTTAATTTGCGAGACGAGTGCTCGACATGGTTTTAGAGCTAGAAATAGC
*SIR2*-*N329A*	CGTAAACTATTTTTAATTTGGATAATTTAGAACAACGAGCGTTTTAGAGCTAGAAATAGC
*NOP1*-*RFP*	CGTAAACTATTTTTAATTTGGCGGAATAAAGAAATAGATTGTTTTAGAGCTAGAAATAGC

a To generate guide RNA expression cassettes, the oligonucleotides were used to amplify “fragment B” from pADH119. This PCR product was then stitched to “fragment A,” as described previously (31). The underlined portion represents the cut site within the C. albicans genome.

**TABLE 5:** Oligonucleotides used to create repair templates^*a*^

Mutation	Oligonucleotides used to create repair template
*hst1*Δ	Top strand: TATATTCTTATTCTTATCAATTGTTACTAATAACAAATAACAATCAATAGGAGAGCCAAAACAAGATAAAGTGGACAACGAGGACAAAGAGGAAGAGAAA Bottom strand: TTTCTCTTCCTCTTTGTCCTCGTTGTCCACTTTATCTTGTTTTGGCTCTCCTATTGATTGTTATTTGTTATTAGTAACAATTGATAAGAATAAGAATATA
*sir2*Δ	Top strand: TTTCTTTATTATATTGACGTTTCAGTTATTTGAGAGAAATCCTCTAGTAGGTTAAATTAATATTGGTGTCTTTAATGTTTTTCTTTTCAATTCTTTTATA Bottom strand: TATAAAAGAATTGAAAAGAAAAACATTAAAGACACCAATATTAATTTAACCTACTAGAGGATTTCTCTCAAATAACTGAAACGTCAATATAATAAAGAAA
*hst2*Δ	Top strand: ATACGTACACTTCTTACAACAGACTATAACTTTAAACCCGAGACGAGTGCTCGACATGAGGAATGTCTCAACAAAAATATAATATACATTTTCAAGAAAG Bottom strand: CTTTCTTGAAAATGTATATTATATTTTTGTTGAGACATTCCTCATGTCGAGCACTCGTCTCGGGTTTAAAGTTATAGTCTGTTGTAAGAAGTGTACGTAT
*HST1* addback	F: CCCCTCACCCTTTCCTC R: CTTAGCCTAATAACCTTAAACCTAAT
*HST2* addback	F: GATGTTAGTCTCAGAACC R: CCTCAGGTAAAATAATACTAG
*SIR2*-*N329A*	1: TAGTTTATTACATGCATTTTTGAAATTATTACAAGATAAACATAAATTATTACGAAATTA 2: CAATCCTGCACGTTGTTCCAAGTTGTCAATGGCTTGAGTATAATTTCGTAATAATTTATG 3: TAGCAAATGATCCATGACATTGAACTAATTTTTCCAGTTTCAATCCTGCACGTTGTTCCA

a Repair templates for gene deletions were prepared by annealing the two oligonucleotides. Repair templates for gene addbacks were amplified from genomic DNA using the indicated oligonucleotides. The repair template for mutating *SIR2*, was constructed through two successive PCRs, as described in Materials and Methods. F, forward; R, reverse.

The addback strains were generated by incorporating the wild-type *HST1* or *HST2* genes back into the endogenous loci in the deletion strains. The repair templates were generated by amplifying the *HST1* and *HST2* genes, including ∼500 bp upstream and downstream. The guide RNAs targeted the deletion site, using a PAM site that had been incorporated into the deletion repair template.

To tag *SIR2*, *HST1*, *HST2*, or *NOP1*, tagged alleles were first generated on plasmids (Table 1). The tagged alleles were released from the plasmids by restriction digestion, extracted from an agarose gel, and then used for transformation. The *SIR2*-*GFP*, *SIR2*-*MYC*, *HST1*-*GFP*, and *HST2*-*GFP* repair templates were integrated into the corresponding knockout strains, generating strains in which both alleles were tagged. The *NOP1*-*RFP* repair template was transformed into wild-type *NOP1* strain, since *NOP1* is an essential gene. We were only able to generate heterozygous *NOP1*-*RFP* strains, suggesting that the RFP interferes with the function of Nop1. Correct integration of the tag was confirmed by Sanger sequencing, and expression was confirmed by immunoblotting.

To create the catalytically inactive *SIR2* allele, we generated a 140-bp repair template changing AAT(Asn) to GCC(Ala) at amino acid position 329. We also introduced synonymous mutations at positions 331, 332, 333, and 336 to prevent the gRNA from recutting *SIR2* after gene editing. To generate the 140-bp repair template, we first created a 100-bp PCR product using two overlapping oligonucleotides (Table 5). This product was then extended by 40 bp in a second PCR. This repair template was integrated into the Sir2-Myc strain. Integration of the mutations was confirmed by Sanger sequencing, and expression was confirmed by immunoblotting.

The strains and plasmids used in this study are available upon request.

### Immunoblotting.

Immunoblots were performed as previously described (56) with modifications. C. albicans cells were grown to an optical density at 600 nm (OD_600_) of 2.5 to 3.0, and 50 OD cells were used to prepare cell lysate. Cells were lysed by bead beating for 30 min at 4°C in 500 μl of lysis buffer (10% glycerol, 150 mM KCl, 10 mM HEPES [pH 7.9], 1.5 mM MgCl_2_, 0.5 mM dithiothreitol, 5 mg/ml chymostatin, protease inhibitors). Proteins were then denatured, resolved on a 10% acrylamide-SDS gel, transferred to a nitrocellulose membrane, and probed with mouse anti-Myc (Millipore, 05-724), anti-GFP (Roche, 11814460001), or anti-Pgk1 (Invitrogen, 459250) antibodies. Immunoblots were quantified using ImageJ.

### Liquid filamentation assay.

Strains were grown overnight with shaking at 30°C in liquid YPD. Overnight cells (10 OD) were pelleted, washed once with phosphate-buffered saline (PBS [8 g of NaCl, 200 mg of KCl, 1.44 g of Na_2_HPO_4_, and 240 mg of KH_2_PO_4_], adjusted pH to 7.4, and filled to 1 liter with water), and resuspended in 1 ml of PBS. We then inoculated 40 μl of the washed cells into 2 ml of YPD or spider medium in 24-well plates. Plates were incubated for 3 h at 37°C with shaking, and then the cells were imaged on a Zeiss AxioVision microscope at 20× magnification. The cells were scored into three categories: yeast, pseudohyphae, and true hyphae (as shown in Fig. 1A and B). The percentages of each cell type were determined by counting at least 200 cells/strain. The averages and standard deviations of the mean of three independent experiments were calculated.

### RNA isolation and quantitative RT-PCR.

To measure the expression of hyphal-specific genes, cells were harvested before (overnight cells grown in YPD) and after hyphal induction (cells grown in spider medium at 37°C for 3 h in 24-well plates). RNA was isolated as previously described (57) with modification in the cell lysate preparation step: cells were lysed with a bead-beating method for 30 min at 4°C, incubated at 65°C for 20 min, chilled in dry ice until frozen solid, and then centrifuged for 10 min at maximum speed in a microcentrifuge to separate the aqueous phase, which contains the RNA, and the phenol phase.

For qRT-PCR, 5 μg of RNA was DNase treated for 30 min at 37°C using Turbo DNase (Invitrogen) to eliminate genomic DNA. cDNA was synthesized using the DNase-treated RNA as the template with the iScript reverse transcription supermix (Bio-Rad), and qPCR was performed using iQ SYBR green Supermix (Bio-Rad). The qPCR was performed on a CFX384 real-time PCR machine (Bio-Rad). The hypha-specific genes (*HWP1*, *ECE1*, and *ALS3*) and a control gene (*ACT1*) were quantified relative to a standard curve prepared from genomic DNA. The primers are listed in Table 6. RNA from four separate cultures was prepared for each strain. The ratio of the hypha-specific gene to the control gene was determined for each replicate. The ratios for the four replicates were averaged, and this value was normalized to the average ratio in wild-type cells not undergoing hyphal induction. Error bars represent the standard errors of the mean (SEM) of four independent qRT-PCR replicates.

**TABLE 6:** Oligonucleotides used for quantitative PCR

Gene	Sequence(s)^*a*^
*HWP1*	F: GCTGGTTCAGAATCATCCATGC R: AAGGTTCAGTGGCAGGAGCTG
*ECE1*	F: TGCCATTTGTTGTCAGAGCTG R: TAGCTTGTTGAACAGTTTCCAGG
*ALS3*	F: CCACTTCACAATCCCCATC R: CAGCAGTAGTAGTAACAGTAGTAGTTTCATC
*ACT1*	F: TGGTGATGGTGTTACTCACG R: GACAATTTCTCTTTCAGCAC

a F, forward; R, reverse.

### Fluorescence microscopy.

Cells were grown as described for the filamentation assay. After a 3-h incubation in YPD at 37°C, 1 ml of cell culture was harvested, pelleted, and resuspended in 0.5 ml of PBS. To stain DNA, 7.5 μl of the resuspended cells and 7.5 μl of freshly prepared 1 μg/ml DAPI (4′,6-diamidino-2-phenylindole) were mixed on microscope slides and incubated in the dark for 30 min. Images were taken with KEYENCE microscope BZ-X800 series with a 100× magnification.
