# Supplementary figures and images for: Genetic Analysis of Sirtuin Deacetylases in Hyphal Growth of Candida albicans
Source: mSphere. 2021 May 5;6(3):e00053-21. doi: 10.1128/mSphere.00053-21 (PMC8103982; doi:10.1128/mSphere.00053-21)

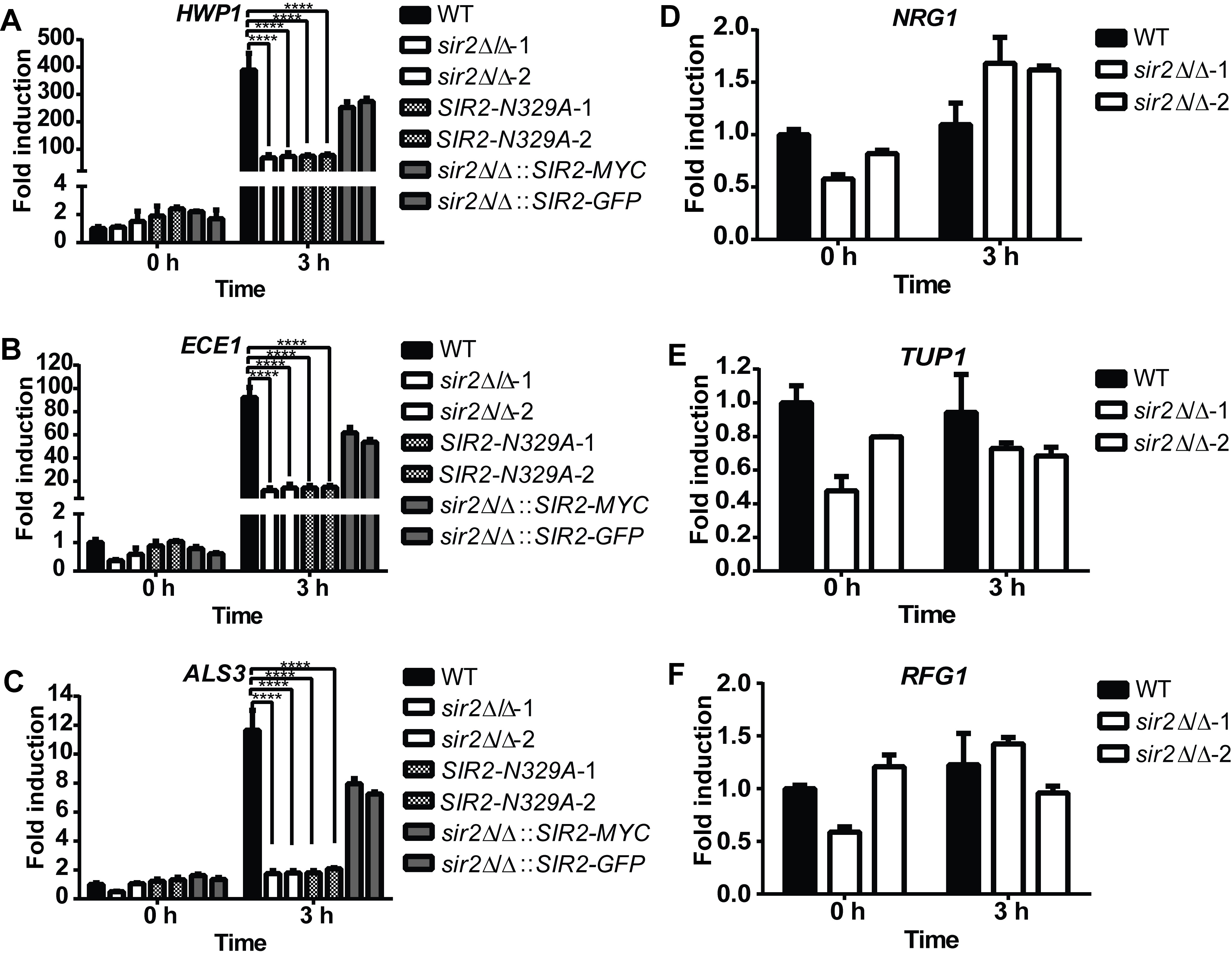

Supplement: FIG S1 [file mSphere.00053-21-sf001.tif]
